# Epidemiological features and spatial–temporal distribution of visceral leishmaniasis in mainland China: a population-based surveillance study from 2004 to 2019

**DOI:** 10.1186/s13071-021-05002-y

**Published:** 2021-10-07

**Authors:** Zhou Guan, Can Chen, Chenyang Huang, Hongwei Zhang, Yiyi Zhou, Yuqing Zhou, Jie Wu, Zhengbin Zhou, Shigui Yang, Lanjuan Li

**Affiliations:** 1 State Key Laboratory for Diagnosis and Treatment of Infectious Diseases, National Clinical Research Centre for Infectious Diseases, Collaborative Innovation Centre for Diagnosis and Treatment of Infectious Diseases, The First Affiliated Hospital, Zhejiang University School of Medicine, Hangzhou, People’s Republic of China; 2 Henan Centre for Disease Control and Prevention, Zhengzhou, People’s Republic of China; 3 National Institute of Parasitic Diseases, Chinese Center for Disease Control and Prevention, Shanghai, People’s Republic of China

**Keywords:** Leishmaniasis, Epidemiology, Mainland China, Incidence

## Abstract

**Background:**

Although visceral leishmaniasis (VL) was largely brought under control in most regions of China during the previous century, VL cases have rebounded in western and central China in recent decades. The aim of this study was to investigate the epidemiological features and spatial–temporal distribution of VL in mainland China from 2004 to 2019.

**Methods:**

Incidence and mortality data for VL during the period 2004–2019 were collected from the Public Health Sciences Data Center of China and annual national epidemic reports of VL, whose data source was the National Diseases Reporting Information System. Joinpoint regression analysis was performed to explore the trends of VL. Spatial autocorrelation and spatial–temporal clustering analysis were conducted to identify the distribution and risk areas of VL transmission.

**Results:**

A total of 4877 VL cases were reported in mainland China during 2004–2019, with mean annual incidence of 0.0228/100,000. VL incidence showed a decreasing trend in general during our study period (annual percentage change [APC] = −4.2564, 95% confidence interval [CI]: −8.0856 to −0.2677). Among mainly endemic provinces, VL was initially heavily epidemic in Gansu, Sichuan, and especially Xinjiang, but subsequently decreased considerably. In contrast, Shaanxi and Shanxi witnessed significantly increasing trends, especially in 2017–2019. The first-level spatial–temporal aggregation area covered two endemic provinces in northwestern China, including Gansu and Xinjiang, with the gathering time from 2004 to 2011 (relative risk [RR] = 13.91, log-likelihood ratio [LLR] = 3308.87, *P* < 0.001). The secondary aggregation area was detected in Shanxi province of central China, with the gathering time of 2019 (RR = 1.61, LLR = 4.88, *P* = 0.041). The epidemic peak of October to November disappeared in 2018–2019, leaving only one peak in March to May.

**Conclusions:**

Our findings suggest that VL is still an important endemic infectious disease in China. Epidemic trends in different provinces changed significantly and spatial–temporal aggregation areas shifted from northwestern to central China during our study period. Mitigation strategies, including large-scale screening, insecticide spraying, and health education encouraging behavioral change, in combination with other integrated approaches, are needed to decrease transmission risk in areas at risk, especially in Shanxi, Shaanxi, and Gansu provinces.

**Graphical abstract:**

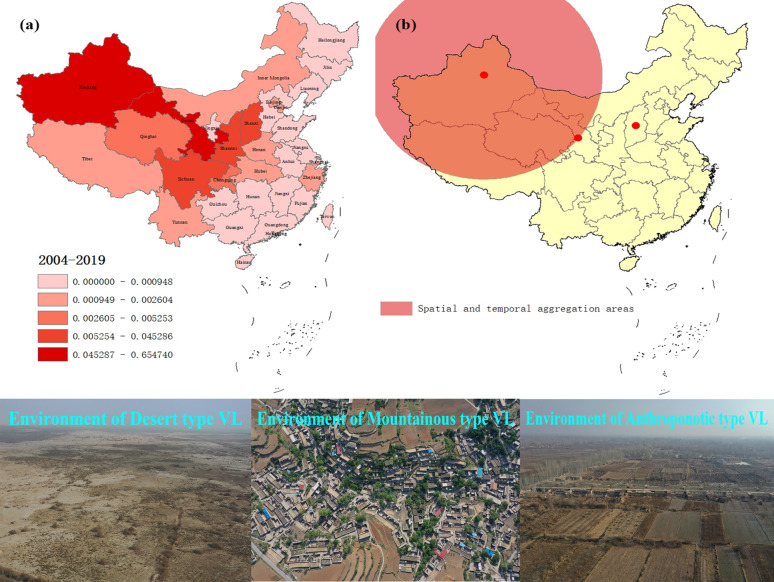

**Supplementary Information:**

The online version contains supplementary material available at 10.1186/s13071-021-05002-y.

## Background

Visceral leishmaniasis (VL), also known as kala-azar, is a life-threatening vector-borne parasitic disease which is caused by trypanosomatid protozoa in the genus *Leishmania* and is transmitted by phlebotomine sand flies [1, 2]. As an important public health issue, VL is widespread, found in over 60 countries worldwide. Every year, an estimated 50,000–90,000 new cases of VL occur around the globe [3]. Based on the associated morbidity and mortality, the World Health Organization (WHO) ranked VL ninth in a global analysis of infectious diseases [4]. In the People’s Republic of China, VL was once seriously endemic in vast regions and has been one of the important public health problems [5].

The fight against VL in China has a history dating back nearly 120 years, since the first confirmed case reported in 1904 [6]. During the following decades, VL became quite rampant in eastern and central China [5, 7, 8]. Due to a lack of effective control measures and inadequate access to treatment, VL then spread across 16 provinces north of the Yangtze River [9], becoming one of the most serious parasitic diseases in China until 1950 [10]. According to a large-scale survey conducted by the government in 1951, approximately 530,000 VL cases were detected, with the incidence ranging from 10/10,000 to 50/10,000 in different areas [11–13]. Subsequently, a strict and comprehensive national control program was put in place, and during the 1950s to the 1980s, VL was largely brought under control [5, 9, 14]. By 1983, the great effort to control VL had enabled the entire plain regions in China to reach the standard threshold of elimination [15]. However, with the development of the economy and increased population mobility, the disease saw a resurgence in western and central China [16], resulting in epidemic outbreaks in 43 counties of six provinces in the 1990s [17]. And since the 2000s, the endemic regions have expanded to more than 80 counties of eight provinces, including the Xinjiang Uygur Autonomous Region, Gansu, Sichuan, Shaanxi, Shanxi, Henan, Hebei, and the Inner Mongolia Autonomous Region [18]. During this historical period, two epidemiological species of VL, namely anthroponotic and zoonotic types, were identified in mainland China [19].

The anthroponotic type (AVL), whose transmission cycle is from human to human, is caused by *Leishmania donovani* [5]*.* This type was once seriously endemic in the plain areas of eastern and central China [20]. After the control program was established, the disease was remarkably controlled in the regions mentioned above, and is currently distributed in the oases of the plains of Kashi Prefecture, Xinjiang [21]. The zoonotic type, caused by *L. infantum*, involves an animal host as a principal infectious source [12, 22]. This type is further classified into two subtypes, i.e., the mountainous type (MT-ZVL) and the desert type (DT-ZVL), based on their specific ecosystem, vector species, and infectious sources [23, 24]. Transmission of these two types was not completely interrupted during the national control program period [25, 26]. Of these two types, MT-ZVL is currently endemic in mountainous regions of western and central China. The most prominent epidemic area of DT-ZVL is located in the desert regions in southern Xinjiang [18]. These three types of VL exhibit substantial differences in epidemiological characteristics, i.e., geographical and landscape features, ecosystem, vector species, and infectious sources [23], and therefore require targeted and timely control measures to efficiently allocate the relatively scarce resources.

To better inform control measures, studies on the trend, variation, and spatial–temporal characteristics of VL are necessary. Although previous studies on VL in China described VL-associated epidemic and mortality estimates, these works were short-term in nature or the results are now outdated. Therefore, we conducted a large-scale epidemiological study and investigated the distribution characteristics of VL by time, region, and population in mainland China from 2004 to 2019. The trend and variation in VL were analyzed using Joinpoint regression. The surveillance data at the provincial level were analyzed by exploratory spatial analysis. This study aimed to explore the trend, variation, and spatial–temporal clustering of VL, which will provide epidemiologists and governments with significant information for prevention and control strategies.

## Methods

### Data sources and study area

**Fig. 1:**
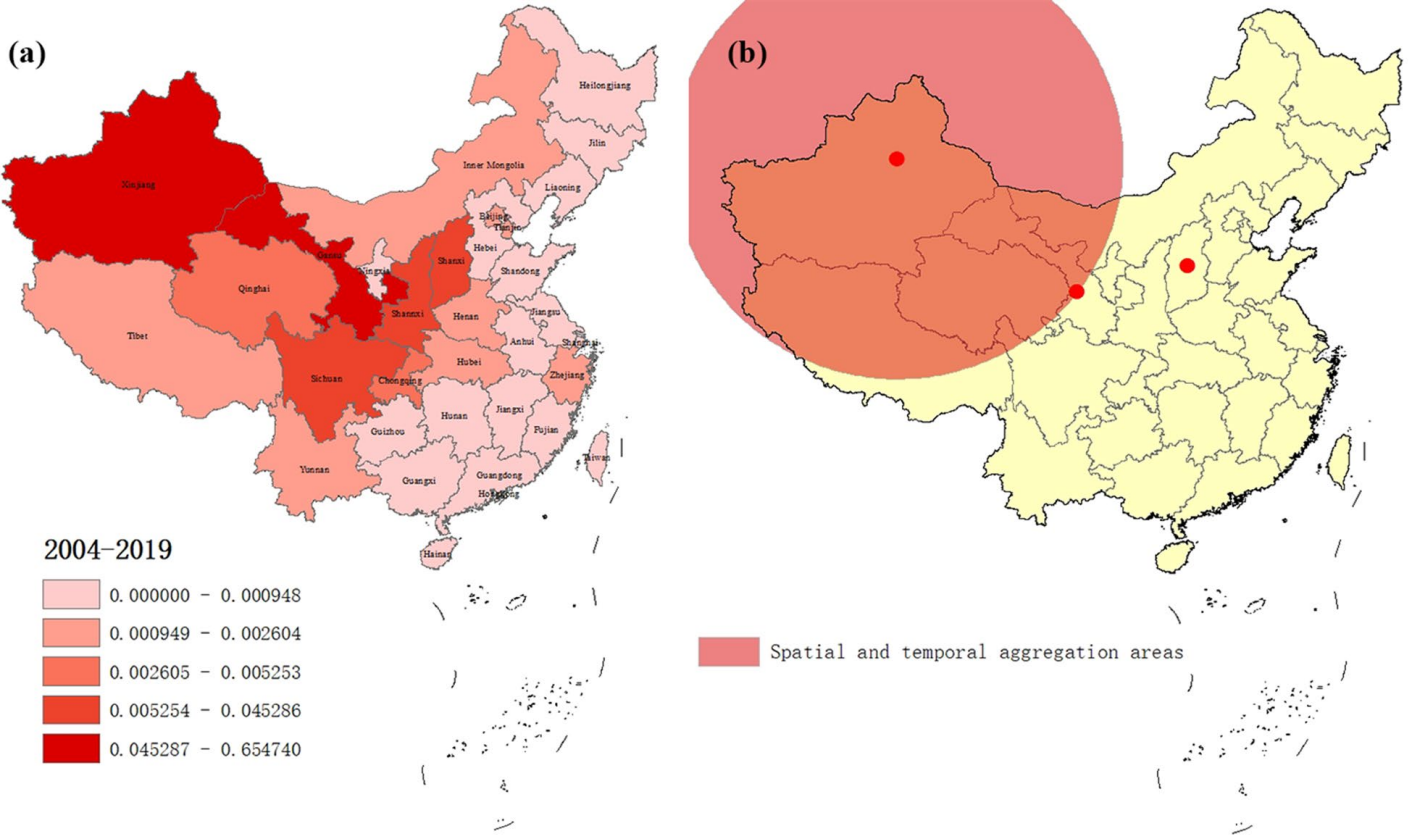
Spatial cluster analysis of visceral leishmaniasis incidence in mainland China. **a** Average annual incidence of visceral leishmaniasis per 100,000 people in 31 Chinese provinces from 2004 to 2019. **b** Spatial cluster analysis of visceral leishmaniasis cases in mainland China. The circle on the map represents the aggregation area for visceral leishmaniasis cases in mainland China from 2004 to 2019. The first-level spatial–temporal aggregation area covered two provinces in northwestern China with gathering time from 2004 to 2011. The secondary spatial–temporal aggregation area was detected in central China with gathering time of 2019

Incidence and mortality data for VL from 2004 to 2017 were provided by the Public Health Sciences Data Center of China, which is a component of the national scientific data sharing platform of the national science and technology infrastructure in China. Surveillance data for 2018–2019 were obtained from annual national endemic status reports on VL [21, 27], whose data source was the National Diseases Reporting Information System (NDRIS) operated by the Chinese Center for Disease Control and Prevention. On January 1, 2004, the national notifiable infectious disease management system was established, which covered a population of about 1.3 billion people from 31 provinces and regions in mainland China. VL is one of the compulsorily notifiable infectious diseases in this surveillance system. The research area of this study covered 31 provinces and regions of mainland China, including eight VL epidemic provinces and 23 non-epidemic provinces. The location of each province is shown in Fig. 1a.

### Data extraction

VL data were collected for the period from January 1, 2004, to December 31, 2019, including the number of cases and deaths, the incidence and mortality, and patient information (age, date of infection, and infection region). In order to assess the epidemiological characteristics and spatial–temporal distribution of VL, data in our study period were stratified by 31 provinces. We also divided this time span into two periods for analysis, i.e., 2004–2017 and 2018–2019, because the national program of fighting against poverty was implemented at an unprecedentedly higher and deeper level beginning in 2018. During the following period, residential environmental and sanitary conditions in rural areas were improved considerably, for example through the large-scale replacement of earthen walls in residential housing with concrete walls in epidemic areas, which effectively reduced sand fly breeding areas. We therefore divided the study period from 2018 to explore more information on changes in the VL epidemic status.

### Quality control

VL cases were required to meet the criteria issued by the Ministry of Health in China. According to the law of prevention and control of infectious disease, clinicians must complete a standardized infectious disease card if they identify any probable, clinical, or laboratory-confirmed VL case. Once receiving an infectious disease card, local epidemiologists perform a field investigation using a standard form [28].

### Statistical analysis

The incidence and incidence rate ratio of VL were calculated at the national and provincial levels, respectively (Table 1). Joinpoint regression software (version 4.8.0.1, National Cancer Institute) was applied to assess the trends in VL incidence. The annual percentage change (APC) and average annual percentage change (AAPC), along with their 95% confidence intervals (95% CI), were calculated for each trend segment. A *t*-test was performed to assess whether an APC or AAPC value increased or decreased significantly. We analyzed the three types of VL endemic in China according to their distinct epidemic provinces. The differences in seasonal patterns and age structure between the two study periods were examined by the Chi-square test using SAS version 9.4 software (SAS Institute Inc./RTI, Cary, NC, USA).

**Table 1:** Time trend analysis of visceral leishmaniasis for eight epidemic provinces in China, 2004–2019

Areas	Number of cases 2004–2019	Number of deaths 2004–2019	Annual mean incidence (per 100,000 people)			Incidence rate ratio	Segmented period			Entire period	
			2004–2019	2004–2017	2018–2019		Period	APC (95% CI)	Trend	AAPC (95% CI)	Trend
Gansu	1605	3	0.3856	0.4115	0.2045	0.4970	2004–2009	13.7479 (− 1.1359, 30.8723)	Stable	− 3.8924 (− 8.6458, 1.1084)	Stable
							2009–2019	− 11.6584* (− 15.8681, − 7.2382)	Decrease		
Hebei	8	0	0.0007	0.0005	0.0020	4.0000	2004–2019	89.4744* (20.1610, 198.7704)	Increase	89.4744* (20.1610, 198.7704)	Increase
Henan	19	0	0.0012	0.0007	0.0052	7.4286	2004–2019	118.0616* (43.1933, 232.0749)	Increase	118.0616* (43.1933, 232.0749)	Increase
Inner Mongolia	10	0	0.0026	0.0030	0.0000	0.0000	2004–2019	− 48.6959* (− 70.2517, − 11.5207)	Decrease	− 48.6959* (− 70.2517, − 11.5207)	Decrease
Shanxi	144	0	0.0244	0.0109	0.1195	10.9633	2004–2019	76.9637* (20.9169, 158.9890)	Increase	76.9637* (20.9169, 158.9890)	Increase
Shaanxi	136	0	0.0223	0.0144	0.0775	5.3819	2004–2019	25.3849* (19.9419, 31.0750)	Increase	25.3849* (19.9419, 31.0750)	Increase
Sichuan	593	5	0.0453	0.0496	0.0150	0.3024	2004–2010	8.1144 (− 4.8372, 22.8286)	Stable	− 8.6387* (− 13.8294, − 3.1353)	Decrease
							2010–2019	− 18.3390* (− 23.7769, − 12.5132)	Decrease		
Xinjiang	2250	3	0.6547	0.7423	0.0421	0.0567	2004–2016	− 3.0432 (− 16.6773, 12.8219)	Stable	− 22.7750* (− 40.0054, -0.5961)	Decrease
							2016–2019	− 68.9203 (− 91.3710, 11.9422)	Stable		
Overall	4877	13	0.0228	0.0243	0.0123	0.5062	2004–2019	− 4.2564* (− 8.0856, − 0.2677)	Decrease	− 4.2564* (− 8.0856, − 0.2677)	Decrease

*APC* annual percentage change, *AAPC* average annual percentage change

*P value < 0.05

The global and local spatial autocorrelation of VL incidence were estimated by GeoDa (version 1.12.1). Global spatial autocorrelation analysis revealed whether a clustering or dispersion existed according to the value of Moran’s *I*. A positive Moran’s *I* value indicated that clustering was present, while a negative Moran’s *I* implied dispersion [29]. Local spatial autocorrelation at the provincial level was reflected by the local indicators of spatial association (LISAs). The LISA clustering map involves four patterns: high–high, high–low, low–high, and low–low. The *Z* test was used to examine significant differences.

Spatial–temporal aggregation analysis was performed based on the discrete Poisson model at the provincial level using SaTScan software (version 9.7). The statistical value was the log-likelihood ratio (LLR), with a larger LLR value indicating a more likely gathering area. The relative risk (RR) was also calculated. Finally, the result was visualized through ArcMap software (version 10.2).

## Results

### The trend in VL incidence from 2004 to 2019

**Fig. 2:**
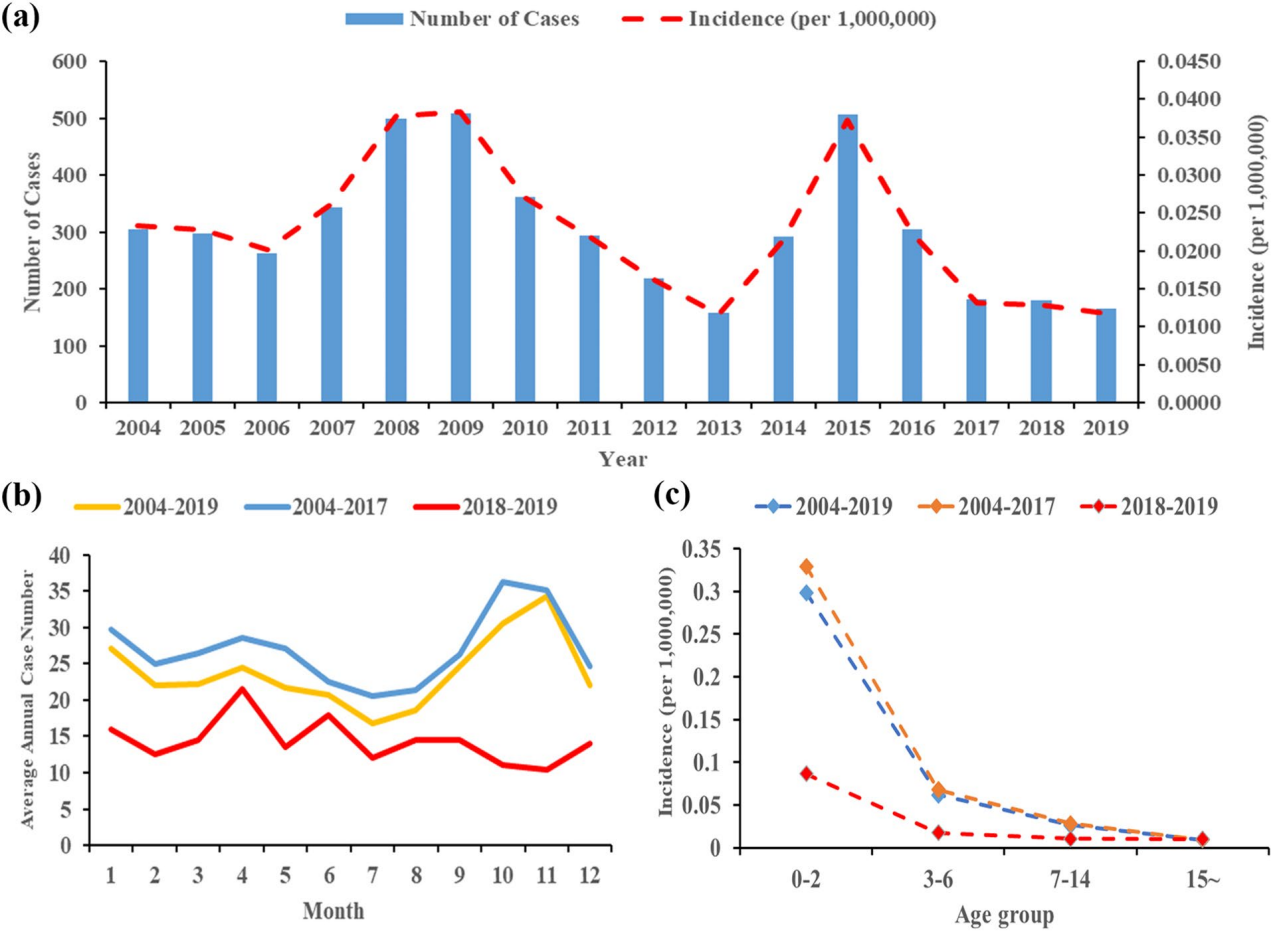
Incidence and number of visceral leishmaniasis cases in mainland China. **a** The incidence per 100,000 people and number of visceral leishmaniasis cases by year from 2004 to 2019. **b** Average annual number of visceral leishmaniasis cases by month among three study periods. **c** The incidence per 100,000 people of visceral leishmaniasis in four age groups among three study periods

A total of 4877 VL cases and 13 deaths were reported in mainland China during the period 2004–2019. The annual mean incidence of VL during this period was 0.0228/100,000 individuals. The initial rising trend started in 2006 (0.0201/100,000), increasing dramatically and peaking in 2009 (0.0383/100,000). The following years witnessed a steady decline until 2013. Subsequently, the trend reversed again and peaked in 2015 (0.0372/100,000), after which the incidence decreased each year until 2019 (Fig. 2a). Joinpoint regression analysis indicated an APC of −4.2564 (95% CI: −8.0856 to −0.2677, *P* = 0.0384), which therefore illustrated a significantly decreasing trend during 2004–2019 (Table 1). Based on the description above, it is clear that despite some enormous fluctuations, there was a steady declining trend in VL incidence in mainland China when evaluating from an overall perspective (Additional file 1: Figure S1 a).

During our study period, VL incidence in the eight epidemic provinces exhibited several distinctive trends. For instance, Shanxi and Shaanxi witnessed significantly increasing trends, with APC values of 76.9637 and 25.3849, respectively (Additional file 1: Figure S1 b, c). Shanxi in particular showed a dramatic rise from 2015, and reached a peak in 2019 with the most cases reported. Gansu and Sichuan both showed an initial nonsignificant increasing trend over a relatively short period, followed by a significantly declining trend, and eventually reaching a relatively low level until 2019 (Additional file 1: Figure S1 d, e). Despite some enormous fluctuations, Xinjiang witnessed an overall downward trend, especially in the last 3 years (Additional file 1: Figure S1 f). Xinjiang had the most VL cases reported in 11/16 years, while after the decline, only three cases were reported in 2019. Hebei, Henan, and Inner Mongolia had relatively few VL cases reported, but showed two distinctive patterns. Hebei and Henan showed increasing trends, especially in the last 4 years, while Inner Mongolia displayed an overall decreasing trend, with no VL cases reported from 2013 onward. The incidence rate ratio ranged from 0.0567 in Xinjiang to 10.9633 in Shanxi, which implies that differences in annual VL incidence existed not only among the eight epidemic provinces, but also between the two study periods (Table 1).

### The seasonal pattern and age structure of VL cases

VL can occur throughout the year, but it displayed an epidemic pattern of a semiannual peak during 2004–2019. The seasonal pattern analysis showed that VL typically occurred from March to May as the first peak, followed by a second peak between October and November. Among our study periods, the seasonal pattern in 2004–2017 was similar to that in 2004–2019. On the contrary, a significant change was observed in 2018–2019, where the epidemic peak in October to November disappeared, leaving only one peak from March to May (Fig. 2b). The VL cases reported in March to May and October to November differed significantly between the two study periods (*χ*^2^ = 14.1002, *P* = 0.0002).

Figure 2c shows that VL incidence was highest in the 0–2 age group, and decreased with age. This downward trend was considerable during 2004–2017, while it was relatively slight in 2018–2019. This was related to the reduction in VL incidence among the 0–2, 3–6, and 7–14 age groups during 2018–2019, while the 15~ group increased slightly. VL cases in those aged 15 years and older accounted for 31.31% of total cases in 2004–2017, but 64.08% during 2018–2019. A significant difference in VL cases was detected in the 0–2 and 15~ age groups between the two study periods (*χ*^2^ = 124.6443, *P* < 0.0001).

### Geographical distribution characteristics of VL

The results of VL geographical distribution showed that the annual mean incidence ranged from 0.0007/100,000 to 0.6547/100,000 in different provinces (Table 1). In the map, the provinces with significantly high incidence were predominantly located in northwestern and central China, including Xinjiang, Gansu, Sichuan, Shanxi, and Shaanxi (Fig. 1a). Among them, Xinjiang and Gansu possessed considerably higher incidence, with the value of 0.6547/100,0000 and 0.3856/100,0000, respectively (Table 1).

When evaluating the two study periods separately, substantial differences can be seen. During 2004–2017, VL cases were mainly reported from northwestern China, including Xinjiang (0.7423/100,0000), Gansu (0.4115/100,0000), and Sichuan (0.0496/100,0000). In contrast, the provinces with higher incidence in 2018–2019 were located in central China, including Gansu (0.2045/100,0000), Shanxi (0.1195/100,0000), and Shaanxi (0.0775/100,0000) (Table 1).

### Spatial autocorrelation analysis

The global spatial autocorrelation analysis indicated that positive spatial clustering existed from 2004 to 2018 (Table 2). Moran’s scatter diagram displays the spatial clustering of epidemic provinces, where the plots in the first quadrant represent high observation value and short distance. Gansu remained in the first quadrant over the whole study period, while Xinjiang was present from 2004 to 2018, and disappeared in 2019. Sichuan existed in the first quadrant in 8/16 years. Shaanxi and Shanxi were present in the first quadrant from 2017 and 2018 onward, respectively (Table 2).

**Table 2:** Spatial cluster analysis of national visceral leishmaniasis incidence, 2004–2019

Year	Moran’s *I*	*Z*	*P*	Correlation	First Quadrant
2004	0.1179	2.5711	0.0340	Positive	Gansu, Xinjiang
2005	0.1459	2.5695	0.0360	Positive	Gansu, Xinjiang
2006	0.1901	2.5831	0.0360	Positive	Gansu, Sichuan, Xinjiang
2007	0.2243	2.7479	0.0360	Positive	Gansu, Sichuan, Xinjiang
2008	0.1302	2.5849	0.0340	Positive	Gansu, Xinjiang
2009	0.1374	2.5758	0.0340	Positive	Gansu, Xinjiang
2010	0.2005	2.5709	0.0360	Positive	Gansu, Sichuan, Xinjiang
2011	0.1554	2.5605	0.0320	Positive	Gansu, Sichuan, Xinjiang
2012	0.1775	2.9494	0.0180	Positive	Gansu, Sichuan, Xinjiang
2013	0.1344	2.6997	0.0220	Positive	Gansu, Sichuan, Tibet, Xinjiang
2014	0.1488	2.5739	0.0340	Positive	Gansu, Xinjiang
2015	0.0280	2.4447	0.0240	Positive	Gansu, Xinjiang
2016	0.1058	2.5042	0.0360	Positive	Gansu, Xinjiang
2017	0.1720	2.4241	0.0390	Positive	Gansu, Shaanxi, Sichuan, Xinjiang
2018	0.1187	1.7914	0.0630	Positive	Gansu, Shanxi, Shaanxi, Sichuan, Xinjiang
2019	0.0846	1.0875	0.1330	No correlation	Gansu, Shanxi, Shaanxi

**Fig. 3:**
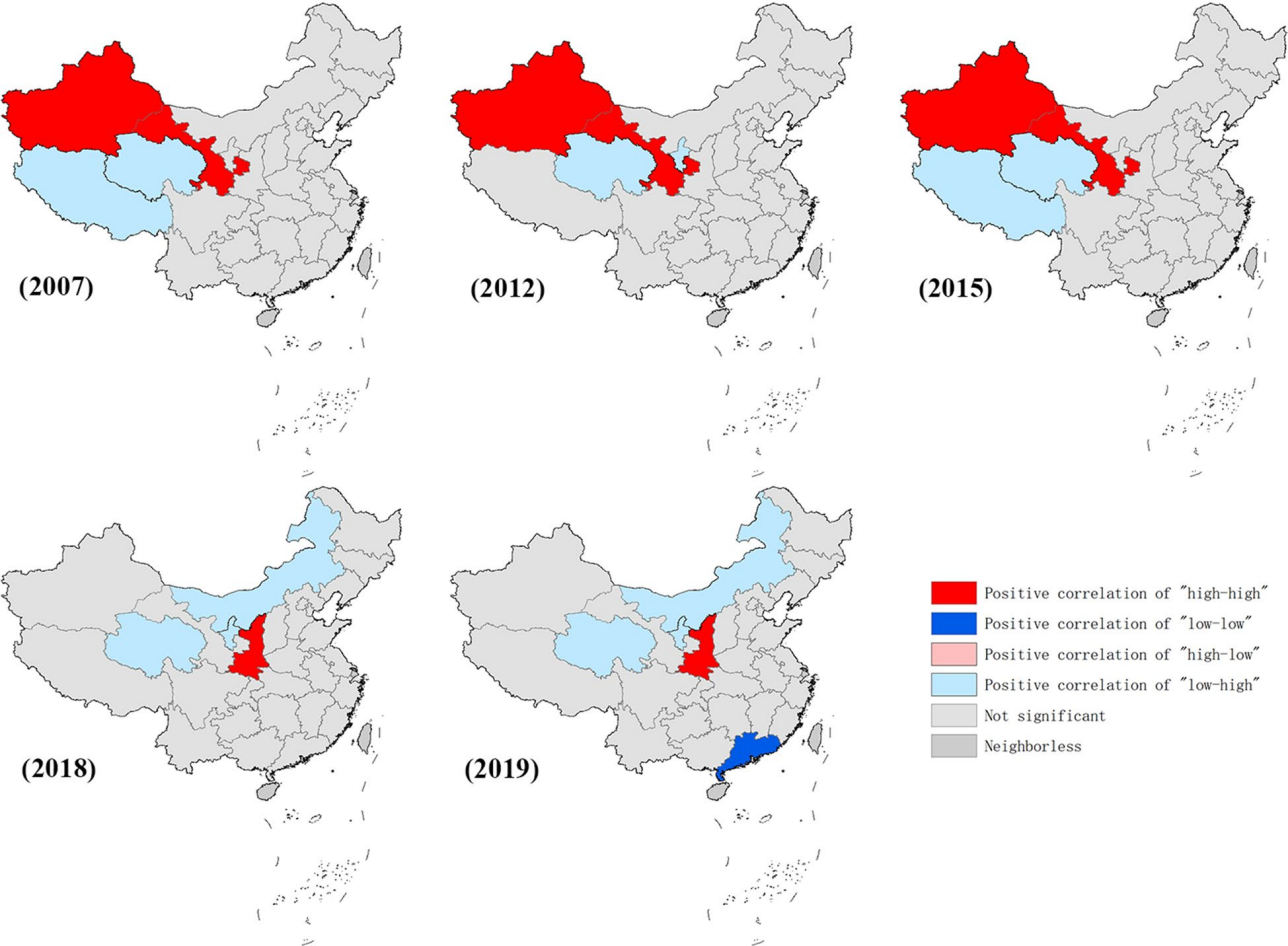
Spatial autocorrelation analysis of visceral leishmaniasis incidence in mainland China in five representative years

Local spatial autocorrelation analysis was subsequently performed at the provincial level, and the results are displayed in the form of a LISA clustering graph. The high–high aggregation showed dynamic changes in VL incidence. In general, the clustering areas were mainly located in northwestern China (including Gansu and Xinjiang) from 2004 to 2016, and then shifted to central China (including Shaanxi) from 2018 to 2019 (Fig. 3).

### Spatial–temporal aggregation analysis

Two spatial and temporal aggregation areas were detected within our study period (Fig. 1b). The first one covered two epidemic provinces in northwestern China, including Gansu and Xinjiang, with the gathering time from 2004 to 2011. During this period, the actual case numbers reported in these areas was 2392, which was significantly higher than expected (321.94). The relative risk (RR) of VL infection in this region was very high, with a value of 13.91 (LLR = 3308.87, *P* < 0.001). The secondary aggregation area was detected in Shanxi province of central China, with a gathering time of 2019. The actual number of cases in this region was 51, while the expected number was 31.87 (RR = 1.61, LLR = 4.88, *P* = 0.041).

## Discussion

VL holds significant importance for global public health, with broad distribution throughout the world, leading to substantial morbidity and mortality each year [30, 31]. Because of the diversity and complexity of VL transmission, timely VL-associated epidemic estimates and assessments among a large-scale population over a long period are necessary to inform a country’s preventative and control measures. In this study, we explored the epidemic trend and spatial–temporal distribution characteristics of VL cases in mainland China over a 16-year study period. The results revealed a declining trend in China from 2004 to 2019. We also found several distinct endemic patterns in different provinces. The northwestern regions, including Gansu, Sichuan, and especially Xinjiang, experienced a decreasing trend in general, while the central areas, including Shanxi and Shaanxi, witnessed an increasing trend, especially in recent years. Significant spatial–temporal aggregation areas of VL were detected during our study period. The clustering regions were initially distributed in northwestern China, and then shifted to central China in the last 2 years. The epidemiological feature analysis indicated that there were significant changes in the seasonal patterns and age structure of VL cases. The changes found in our study could inform the design of targeted preventative and control strategies against VL in China.

VL incidence in China during 2004–2019 exhibited substantial fluctuation, with peaks observed in 2008–2009 and 2015. This trend was basically in accord with that of Xinjiang. As one of the most highly affected VL endemic areas in China, Kashi Prefecture in Xinjiang witnessed two outbreaks, in 2008–2009 and 2015. The case numbers in Kashi Prefecture were very high during these two periods and therefore significantly affected the trend for mainland China [32]. However, according to Joinpoint regression analysis, an overall downward trend was detected. This might be related to the decreasing trends occurring in Gansu, Sichuan, and especially Xinjiang, which were previously the most seriously affected epidemic provinces.

Among the eight epidemic provinces, substantially different trends were discovered. Joinpoint regression indicated that Shanxi and Shaanxi witnessed significantly increasing trends. This is consistent with previous studies, which also revealed a resurgence of MT-ZVL in Shanxi and Shaanxi provinces and showed that cases of infection clustered mainly in the Shanxi-Shaanxi border areas and eastern Shanxi province [33]. This resurgence might be associated with several factors. Firstly, Shanxi and Shaanxi are both MT-ZVL endemic provinces, with their mountainous areas being the epidemic regions. Canine animals are the main reservoir host and infectious source of MT-ZVL. Thanks to the control program conducted in the 1950s, the number of stray and domestic dogs in these areas declined considerably. However, VL transmission in these regions was not completely interrupted. With the rapid economic and societal development, a growing number of rural residents flooded the cities seeking better job opportunities. As a result, young children and elderly people were left behind, and they began keeping dogs for safety reasons. Combined with other suitable transmission factors, namely vectors, climate, and environment, a resurgence in VL therefore occurred in these two provinces. Secondly, the predominant control strategy in MT-ZVL epidemic regions was patient treatment. However, this measure cannot effectively reduce VL incidence. Thirdly, although the application of insecticide-impregnated dog collars had been proven to be effective in other countries [34], there are some difficulties in large-scale implementation in China. Fourthly, spraying of insecticides had limited effects on vector control because of the exophilic behavior of the sand fly in MT-ZVL endemic areas. Additionally, Shanxi and Shaanxi provinces border Hebei and Henan, where VL cases also re-emerged. This might be related to latent infection or imported infectious sources, which need to be further investigated. On the contrary, despite an initial upsurge, VL incidence in Gansu and Sichuan experienced significantly decreasing trends. Xinjiang was initially the most heavily affected VL epidemic region in China, and remained an epidemic province for AVL and DT-ZVL, with AVL distributed in the oases of the plains of Kashi Prefecture and DT-ZVL located in the desert regions in southern Xinjiang. However, in the last 3 years, VL incidence in Xinjiang declined dramatically, with only three cases reported in 2019. In these three provinces, the majority of VL cases were mainly concentrated in a few counties, all of which are located in underdeveloped areas, where almost no funds were previously reserved for disease prevention [35]. The reduction revealed in this study might be thanks to the national program of fighting against poverty. With strong support from the government, the residential environmental and sanitary conditions in rural areas were improved considerably. For instance, a large-scale project was undertaken in epidemic areas to replace earthen walls in residential housing with concrete walls, which effectively reduced the sand fly breeding areas and therefore reduced the contact frequency between sand flies and humans. However, several challenges still exist in these regions. Vectors are widely distributed in this area and difficult to control. Latent infection and wild animal infectious sources might still exist in these regions, which makes VL resurgence possible. The results of spatial autocorrelation and spatial–temporal aggregation analysis indicated that the cluster areas of VL shifted from northwestern China (including Gansu, Xinjiang, and Sichuan) to central China (including Shaanxi and Shanxi), which is in accord with that of Joinpoint regression. It also coincides with a previous study in mountainous areas, which revealed a gradual shift in VL cluster areas from southern Gansu and northern Sichuan province to the Shanxi-Shaanxi border areas and eastern Shanxi province [33]. These changes found in our study illustrate that MT-ZVL endemic regions in central China require more attention, and integrated control measures need to be implemented.

Significant changes in seasonal patterns were revealed in 2018–2019, which might be predominantly associated with the decline that occurred in Xinjiang. DT-ZVL was previously seriously endemic in Xinjiang, with the epidemic peak typically seen in October–November. However, after the reduction in Xinjiang, these peaks have disappeared in recent years, leaving only one peak in March to May. Children aged 0–2 remained the most at-risk population throughout our study period, although the age structure of VL cases changed in 2018–2019. This might be associated with the following. Firstly, the most at-risk population for DT-ZVL was children aged 0–2. With the decline of DT-ZVL in Xinjiang, case numbers in this age group dropped significantly. Secondly, among MT-ZVL cases, the proportion of children aged 0–2 has also decreased in recent years, probably because the age structure of rural residents changed in endemic regions [12, 36]. These findings indicate that attention should be focused on all age groups when performing preventative measures, and comprehensive control strategies are required before and during March to May.

Based on the trends and variations in VL prevalence in mainland China, some measures should be taken to achieve further reductions: (1) Controlling infectious sources. During the epidemic seasons, large-scale screening of infectious sources should be conducted among at-risk population in endemic areas. Once cases are diagnosed, medical treatment should be provided immediately. Meanwhile, measures including killing infected dogs and restricting domestic dog-keeping should also be taken. (2) Reducing VL transmission. Insecticide should be sprayed in vector habitats before their breeding season and during transmission season to reduce the density of sand flies. (3) Protecting vulnerable populations. Health education should be popularized to encourage residents to use insecticide-impregnated nets and sand fly repellent incense, reduce outdoor activities, avoid traveling to epidemic areas, and prevent sand fly bites. (4) Strengthening surveillance. The most effective approach for tracking the changes in VL prevalence is facility-based surveillance in the outpatient or hospital setting. (5) Improving the diagnostic capacity of clinicians. In non-epidemic areas, there is a general lack of awareness about VL diagnosis among clinicians, which could lead to serious outcomes among patients and the spread of VL. Therefore, training programs among clinicians are required to achieve early detection and treatment.

Despite the many important discoveries revealed in this study, it is not without limitations. Firstly, there are three types of VL endemic in eight provinces in mainland China, which demonstrate substantial differences in epidemiological characteristics. However, because of the limited surveillance data collected by the NDRIS of China, at present there is a lack of information on precise epidemiological type and indigenous/imported classification of individual VL cases within mainland China. Therefore, it was not possible to conduct trend and spatial cluster analysis stratified by VL epidemiological type and indigenous/imported classification in this study. However, based on the distinctive endemic regions of these three types of VL, we analyzed and described them according to their respective epidemic provinces. We hope this paper can provide broader insight into the epidemic trends and spatial–temporal distribution of VL in China. Secondly, for multiple reasons, differentiating the clinical characteristics of VL from those of similar diseases is difficult, which might result in some VL cases being misdiagnosed. Additionally, some deaths might be classified into other categories, which may underestimate the true level of VL incidence and mortality. Ongoing studies in this area will hopefully raise awareness among clinicians and the public regarding VL, which might alleviate this situation. This study was able to identify the endemic trends of VL in different regions and identify its spatial–temporal clustering over an extended period, which revealed that, although decreased at the national level, VL incidence saw a significantly increasing trend in Shanxi and Shaanxi provinces. The focus of prevention and control efforts should therefore be shifted from northwestern to central China, especially Shanxi, Shaanxi, and Gansu provinces. Future investigations are needed to collect information on epidemiological type and indigenous/imported classification of individual VL cases. Additionally, stratified analysis of trends and spatial clusters should be performed at the city or county level in relatively small regions to further explore epidemic patterns of VL and provide timely information on prevention and control.

## Conclusions

In summary, our large-scale epidemiological study demonstrated that the annual incidence of VL in mainland China decreased over the period 2004–2019. Among epidemic provinces, Gansu, Sichuan, and especially Xinjiang were initially heavily endemic regions, while VL incidence decreased considerably in later years. In contrast, Shaanxi and Shanxi witnessed a significantly increasing trend in 2017–2019. Significant spatial and temporal aggregation patterns were detected, which also revealed that the cluster areas shifted from northwestern to central China. Changes in seasonal patterns and age structure were observed during our study period. The epidemic peak of October to November disappeared in 2018–2019, leaving only one peak in March to May. Efforts are needed to improve large-scale screening, insecticide spraying, and health education encouraging behavioral change in combination with other integrated approaches to decrease the transmission risk in vulnerable locations, especially in Shanxi, Shaanxi, and Gansu provinces. Further studies stratified by VL epidemiological type and indigenous/imported classification should be performed at the city or county level to further explore the endemic patterns of VL in different regions and better inform control strategies.

## Supplementary Information

**Additional file 1: Figure S1.** Joinpoint regression analysis of visceral leishmaniasis incidence of mainly epidemic provinces in mainland China from 2004 to 2019.

## Abbreviations

VL: Visceral leishmaniasis
MT-ZVL: Mountainous type zoonotic visceral leishmaniasis
DT-ZVL: Desert type zoonotic visceral leishmaniasis
AVL: Anthroponotic visceral leishmaniasis
OR: Odds ratio
95% CI: 95% Confidence interval

## Declarations

### Availability of data and materials

Data supporting the conclusions of this article are included within the article.
