# Supplementary figures and images for: Epidemiological features and spatial–temporal distribution of visceral leishmaniasis in mainland China: a population-based surveillance study from 2004 to 2019
Source: Parasit Vectors. 2021 Oct 7;14:517. doi: 10.1186/s13071-021-05002-y (PMC8499449; doi:10.1186/s13071-021-05002-y)

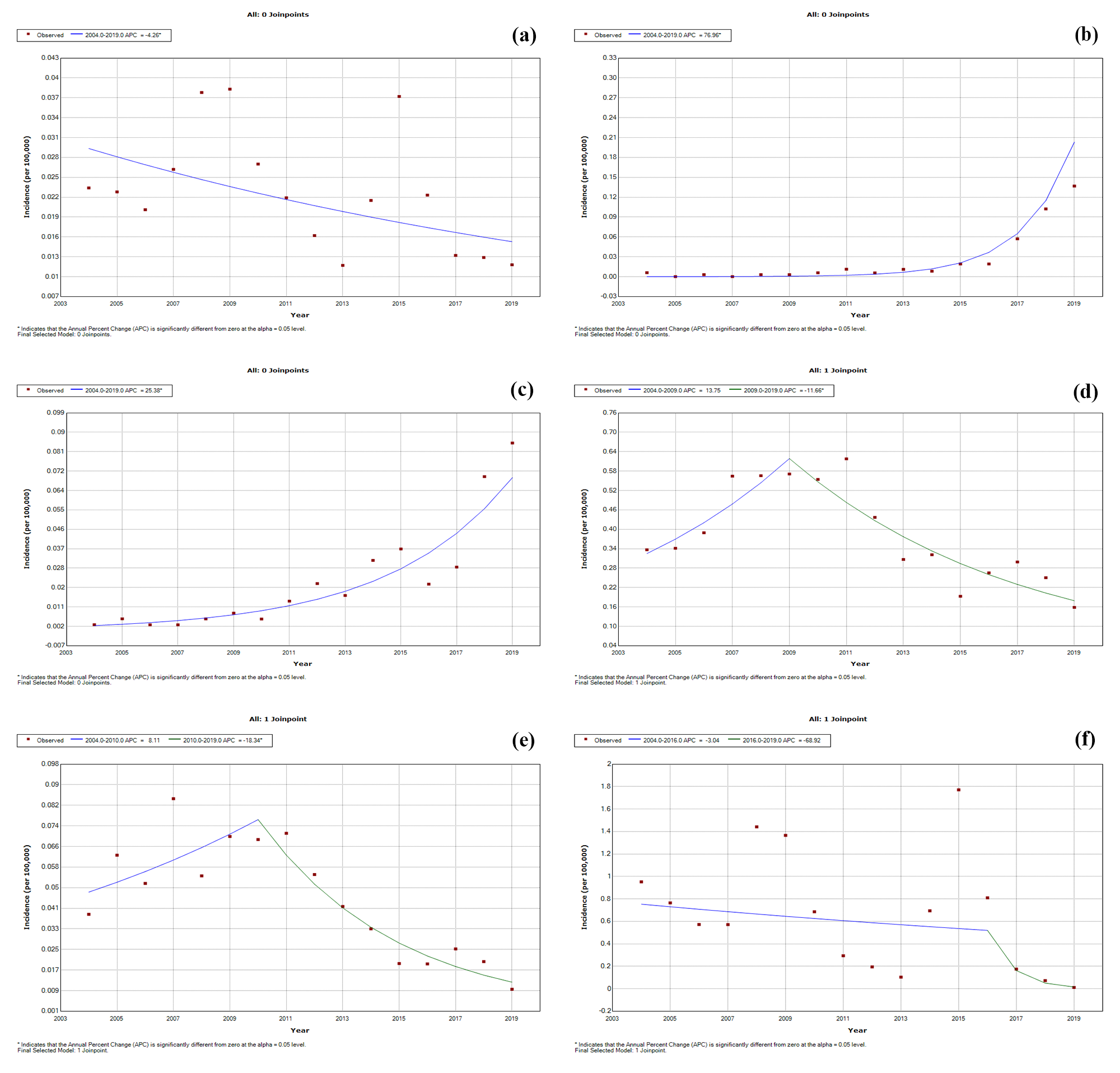

Supplement: Supplementary file 1 — Additional file 1: Figure S1. Joinpoint regression analysis of visceral leishmaniasis incidence of mainly epidemic provinces in mainland China from 2004 to 2019. [file 13071_2021_5002_MOESM1_ESM.tif]
